# Association of microtubule-based processes gene expression with immune microenvironment and its predictive value for drug response in oestrogen receptor-positive breast cancer

**DOI:** 10.3389/fimmu.2025.1608991

**Published:** 2025-07-30

**Authors:** Zhenfeng Huang, Minghui Zhang, Nana Zhang, Mengyao Zeng, Yao Qian, Meng Zhu, Xiangyan Meng, Ming Shan, Guoqiang Zhang, Feng Liu

**Affiliations:** ^1^ Department of Breast Surgery, Harbin Medical University Cancer Hospital, Harbin, Heilongjiang, China; ^2^ Department of Oncology, Chifeng Municipal Hospital, Chifeng, China; ^3^ Department of Medical Training, Aimiker Technology Development Co., Ltd., Nanjing, Jiangsu, China; ^4^ Department of Pathology, Harbin Medical University, Harbin, Heilongjiang, China

**Keywords:** ER+ breast cancer, microtubule-based processes, immune microenvironment, drug resistance, prognosis

## Abstract

**Objective:**

The development of acquired endocrine resistance and reduced chemosensitivity in oestrogen receptor-positive (ER+) breast cancer presents significant challenges. Microtubule-based process-related genes (MBPRGs) play essential biological roles in the cell cycle and the development of migration. This study aimed to establish a novel prognostic signature based on MBPRGs to improve patient outcomes and offer additional treatment options for those with ER+ breast cancer.

**Methods:**

Clinical data along with relevant RNA information with ER+ breast cancer were sourced from The Cancer Genome Atlas and the Molecular Taxonomy of Breast Cancer International Consortium. Consensus clustering was subsequently utilised to identify new molecular subgroups. Evaluations of the tumour immune microenvironment and immune status of these subgroups were performed via ESTIMATE, CIBERSORT, MCP, and ssGSEA. Additionally, functional analyses were conducted to investigate the underlying mechanisms involved. Prognostic risk models were developed via random forest, support vector machines and the least absolute shrinkage and selection operator algorithm. Single-cell analysis revealed differences in the expression levels of key genes among various cell types. Western blotting was used to measure protein levels in breast cancer cell lines. Immunohistochemical staining was used to assess protein expression in paraffin-embedded tissues, and Kaplan–Meier survival curves were generated to evaluate survival differences between the high- and low-expression groups of key genes. Transwell and cell viability assays were used to examine the biological functions of CHORDC1.

**Results:**

Two molecular subgroups with significantly different survival outcomes were identified. Longer survival was linked to a high immune score, low tumour purity, a greater presence of immune infiltrating cells, and an overall positive immune status. Risk models derived from MBPRGs exhibited strong potential for predicting survival in patients with ER+ breast cancer. Key genes had elevated protein levels in differentiated breast cancer cell lines, and elevated CHORDC1 expression was linked to a tendency towards a worse outcome in patients with ER+ breast cancer. Silencing CHORDC1 inhibited cell viability and invasion, reducing sensitivity to tamoxifen and paclitaxel *in vitro*.

**Conclusion:**

MBPRG expression is linked to the immune microenvironment and drug resistance in ER+ breast cancer patients, providing a reliable prognostic indicator for this group.

## Introduction

Breast cancer (BC) is one of the most prevalent cancers affecting women globally and represents a significant health concern because of its high rates of morbidity and mortality (1). In the United States, 300,590 new breast cancer cases were documented in 2023, with approximately 80% classified as oestrogen receptor-positive (ER+) (2). The standard treatments for these patients are endocrine therapy and chemotherapy. However, individuals with ER+ breast cancer tend to exhibit lower sensitivity to chemotherapy than those with oestrogen receptor-negative breast cancer (3). While many ER+ tumours initially respond well to antioestrogens, resistance can develop over time, leading to clinical relapses that are often associated with genetic and epigenetic changes that reactivate ER signalling pathways. This highlights the urgent need to identify more effective therapeutic targets (4).

Microtubules, which are tubular structures formed from tubulin proteins, are essential for various cellular functions and are integral components of the cytoskeleton (5). Microtubule-based process-related genes (MBPRGs) play significant roles in key cellular activities, including cell division, motility, maintenance of cell shape, signalling, and intracellular transport (6). Additionally, MBPRGs are involved in cancer-related processes such as mitosis and cellular migration, thereby contributing to tumour progression and metastasis. They also play a role in signalling pathways that are crucial for cancer cell survival, apoptosis, and responses to stress (7–9). A range of microtubule-targeting agents, including taxanes and colchicine, have been developed and are widely used as first-line treatments for cancer, significantly increasing patient survival rates. One study revealed the potential of podofilox, a microtubule destabiliser, as an effective cGAMP-STING signalling pathway enhancer for antitumour activity (10). In addition, microtubule-associated genes have already shown great potential as biomarkers in lung cancer and osteosarcoma (11, 12). The potential of microtubules as therapeutic targets in oncology is well established.

In recent years, genomic expression profiling has emerged as an essential method for predicting survival outcomes in cancer patients. Many studies have concentrated on prognostic signatures associated with factors such as autophagy (13) and hypoxia (14) in breast cancer, especially in subtypes such as triple-negative (15) and HER2-positive cancers (16). Nonetheless, there has been insufficient research examining the prognostic relevance of microtubules in the treatment and outcomes of breast cancer.

This study aimed to analyse differentially expressed MBPRGs using data from The Cancer Genome Atlas (TCGA) to uncover their enriched pathways and potential biological roles. We developed a model comprising three MBPRGs through multiple Cox regression analyses and performed stratified analyses on various subgroups. Furthermore, we employed the Molecular Taxonomy of Breast Cancer International Consortium (METABRIC) datasets for external validation. Our study also explored the associations between the protein expression levels of MBPRGs and different clinicopathological factors and outcomes in patients with ER+ breast cancer. In summary, our results suggest that MBPRGs play important roles in ER+ breast cancer and may act as prognostic biomarkers and therapeutic targets.

## Materials and methods

### Data collection and preliminary processing

Clinical data and RNA sequencing information were retrieved from both the TCGA and METABRIC databases. The RNA profiling data for the TCGA cohort were in the form of FPKMs. METABRIC’s RNA profiling data are in the form of microarray data at log intensity levels. The inclusion criteria were as follows: (a) samples confirmed as ER+ breast cancer; (b) samples with corresponding clinical data and gene expression profiles; and (c) samples that included thorough clinical information, such as survival duration, survival status, age, and treatment protocols. The exclusion criteria included (a) samples from normal tissues; (b) samples lacking complete clinical data; and (c) samples in which more than 50% of the genes lacked expression values. In total, 803 samples from the TCGA database were set aside as the training cohort, while 1,444 samples from the METABRIC database were merged to create the validation cohort. Datasets of 948 MBPRGs were obtained from the MSigDB database (http://www.hmdb.ca).

### Determination of molecular subgroups and evaluation of the tumour immune environment

Initially, a total of 120 genes associated with the survival of ER+ breast cancer patients were identified via univariate Cox regression analysis using the R package “survival”. Consensus clustering was conducted with the R package “ConsensusClusterPlus,” utilising the expression matrix of these 120 genes. The immune and stromal scores for ER+ breast cancer patients from TCGA were determined using the ESTIMATE algorithm (17), which reflects the presence of gene signatures associated with immune and stromal cells. The CIBERSORT algorithm (18) was utilised to assess the relative abundance of tumour-infiltrating immune cells within the tumour samples. The MCP score (19) provides absolute abundance estimates for eight primary immune cell types. The presence of 28 distinct types of infiltrating immune cells in tumour samples was assessed using single-sample gene set enrichment analysis (ssGSEA).

### Enrichment analysis

The R package ‘Limma’ was used to identify differentially expressed genes (DEGs) between the two clusters. The criteria for identifying DEGs were a fold change of at least ±1.5 and a p value of less than 0.05 (20). The “clusterProfiler” R package was used to perform Gene Ontology (GO) and KEGG pathway enrichment analyses on these DEGs, with a significance threshold of P < 0.05. The outcomes were illustrated through histograms, bubble charts, and circular plots created with the “enrichplot” and “ggplot2” R packages. Furthermore, gene set enrichment analysis (GSEA) was performed to explore the distinctions between the clusters using the same dataset.

### Development and verification of the prognostic signature based on MBPRG

Prognostic indicators grounded in MBPRG were devised and authenticated via three machine learning approaches: least absolute shrinkage and selection operator (LASSO) regression (21), random forest (RF) (22), and support vector machines (SVMs) (23). These techniques were employed to pinpoint essential genes from the most prominent gene clusters chosen through univariate Cox regression analysis. LASSO Cox regression allowed us to evaluate variations in regression coefficients for the pertinent genes, with the ideal parameter λ established through 10-fold cross-validation utilising the R package glmnet. In the end, we chose genes on the basis of lambda.min and illustrated coefficient shrinkage for LASSO Cox regression using plots from R packages. The RF approach utilised the “Random Forest” R package to assess the importance of the key genes identified via univariate Cox regression analysis. SVM functions as a supervised machine learning method for tasks involving regression or classification. The SVM method trains a subset of features from various groups to refine the feature set and identify the most important features. The risk score for every patient in the training and validation cohorts was computed via the following formula: risk score = (0.2745 * CHORDC1) + (1.0279 * WNT3A) + (0.4771 * MECP2). Patients were subsequently divided into high-risk and low-risk categories according to the median value. To evaluate the model’s stability and applicability in predicting overall survival (OS), we conducted univariate and multivariate Cox regression analyses, receiver operating characteristic (ROC) curve analysis, and Kaplan–Meier (K–M) analysis (24). Additionally, genomic data from the Genomics of Drug Sensitivity in Cancer database were utilised to predict the chemosensitivity of the enrolled breast cancer patients. The half maximal inhibitory concentration (IC50) values were computed via the “pRRophetic” package to represent the drug response of patients in different risk score categories.

### Development and evaluation of microtubule-based process-related clinicopathologic nomograms

Cox regression models were utilised to identify univariate prognostic features. A new nomogram based on microtubule-related processes was created, incorporating the risk score along with four clinical variables (age, clinical stage, and whether to use chemotherapy or endocrine therapy) using data from the TCGA cohort, utilising the “regplot” and “rms” R packages. Calibration curves for 3-year and 5-year OS were produced to evaluate the precision of our nomograms. To delve deeper into the clinical significance of the risk scores, box plots based on the results of the Wilcoxon test were created to demonstrate the differences in risk scores among various clinicopathological factors.

### Single-cell RNA sequencing analysis

Three ER+ breast cancer (GSM4909299, GSM4909317, and GSM4909319) single-cell RNA sequencing samples were sourced from GSE161529. The Seurat package was subsequently used to refine the scRNA-seq data, selecting cells of higher quality. Normalisation was carried out via the “normalizedata” function in the Seurat R package. Principal component analysis (PCA) was conducted via the “RunPCA” function in Seurat, focusing on the top 2000 genes to reduce data dimensionality. The cells were grouped and characterised with the “FindNeighbors” and “FindClusters” functions at a resolution of 1. Cell type annotation was performed with the singleR package, and the reference dataset was the Human Primary Cell Atlas (25). The resulting clusters were visualised in two dimensions using the “RunTSNE” and “RunUMAP” functions. Additionally, the “Monocle 2” package was used to predict possible lineage differentiation trajectories. We employed the R package CytoTRACE to compute the CytoTRACE score specifically for cancerous cells to forecast their relative differentiation states on the basis of single-cell transcriptomic data (26).

### Cell culture

The human oestrogen receptor-positive breast cancer cell line MCF-7 and T47D, the triple-negative breast cancer cell line SUM-159PT, the HER2-positive breast cancer cell line UACC812, and the normal human epithelial breast cell line MCF-10A were obtained from Procell Life Science & Technology Co., Ltd. (Wuhan, China). MCF-7, T47D, and MCF-10A cells were cultured in DMEM (Gibco) supplemented with 10% FBS (Gibco) and 10 U/mL penicillin–streptomycin (Gibco). SUM-159PT and UACC-812 cells were grown in RPMI 1640 medium (Sigma–Aldrich, St. Louis, MO, USA). All of the cells were cultured at 37°C in an environment containing 5% CO2.

### Western blots

Total protein was isolated from the cell lines using RIPA lysis buffer supplemented with protease inhibitors. Equal quantities (30 μg) of protein samples were separated using 12% and 8% sodium dodecyl sulfate–polyacrylamide gel electrophoresis (SDS–PAGE) and subsequently transferred to polyvinylidene fluoride (PVDF) membranes (Millipore, USA). The membranes were incubated overnight at 4°C with specific primary antibodies. The following day, the membranes were washed and then incubated with secondary antibodies conjugated to horseradish peroxidase. The PVDF membranes were then exposed to enhanced chemiluminescence (ECL) reagent (Meilunbio, CN) to visualise the positive bands. Anti-CHORDC1 antibody (1:4000), anti-WNT3A antibody (1:1000), anti-MECP2 antibody (1:4000), and anti-β-actin antibody (1:5000) were obtained from Proteintech (Wuhan, CN). The protein product of the CHORDC1 has a molecular weight around 37 kDa. However, the molecular weight of WNT3A, MECP2 and β-actin were at 42 kDA, 75 kDa and 42 kDA, respectively.

### Immunohistochemistry

From Harbin Medical University Cancer Hospital, we acquired formalin-fixed, paraffin-embedded tissue sections from 50 individuals with ER+ breast cancer who underwent surgery between March 2013 and June 2022. Following surgery, patients received endocrine therapy and chemotherapy. Disease-free survival (DFS) refers to the duration from diagnosis until the first occurrence of cancer recurrence, the development of a second cancer, or death from any cause. Overall survival (OS) was defined as the time elapsed from the diagnosis of breast cancer to death from any reason. Sample collection and clinicopathologic data were obtained after informed consent and ethics committee approval were obtained. The formalin-fixed and paraffin-embedded sections were deparaffinised with xylene and ethanol, followed by washing with distilled water. The sections were pretreated with EDTA Target Retrieval Solution at 120°C, pH 8.0, for 3 minutes in a pressure cooker, and to inhibit endogenous peroxidase activity, a solution of 3% H2O2 in PBS was applied for 10 minutes. Nonspecific binding was mitigated by incubating the sections with goat serum for 1 hour. Next, the sections were incubated with primary antibodies overnight at 4°C and then incubated with secondary antibodies for 30 minutes at 37°C. The primary antibody used was CHORDC1, while the secondary antibody was goat anti-rabbit IgG. Colour development was achieved through diaminobenzidine (DAB) staining. Two pathologists independently assessed all of the samples without bias and evaluated the percentage of positively stained membranes. Written informed consent was obtained from each patient.

### Transient transfection

siRNAs were obtained from Sangon Biotech Co., Ltd. in Shanghai, PR China. MCF-7 cells were seeded in a six-well plate at approximately 50% confluency, after which the medium was replaced with 1.5 ml of 10% culture medium free from penicillin and streptomycin. A mixture consisting of 200 µl of jetPRIME^®^ buffer, 5 µl of siRNA, and 4 µl of jetPRIME^®^ reagent was added.

### Cell viability assay

Cell proliferation was assessed via the 3-[4,5-dimethylthiazol-2-yl]-2,5-diphenyl tetrazolium bromide (MTT) and 5-ethynyl-2-deoxyuridine (EDU) assay. The cells were plated in 96-well plates in medium supplemented with 10% FBS, with approximately 2000 cells per well, 24 hours posttransfection. To assess cell viability, cultures were stained with the MTT solution four days later. The absorbance was recorded at 492/562 nm with the help of a microplate reader. For EDU experiments, approximately 5×10⁵ cells were collected, enumerated, and placed into 24-well cell culture plates for a 24-hour period. Subsequently, after the introduction of 10 μM EDU (Beyotime, China), the cells were further incubated for an additional 2 hours. Following this, the cells underwent washing with PBS, fixation using 4% formaldehyde for 15 minutes, and permeabilisation with 0.5% Triton X-100 for 10 minutes. Then, the cells were subjected to treatment with 200 μl of 1×Click Additive Solutionl for 30 minutes at room temperature in the absence of light. The DNA was subsequently stained with 200 μl of 1×Hoechst 33342 for 10 minutes under the same conditions. Finally, the cells were visualised using a laser scanning confocal microscope (FV-1000; Olympus), and the number of positive cells in five randomly selected fields was determined.

### Cytotoxicity assay

MCF7 cells were cultured for 24 hours, followed by the addition of varying concentrations of paclitaxel (0, 5, 10, 15, 20, or 25 nM) and tamoxifen (0, 2, 5, 20, 50, or 100 μM) for 48 hours. Next, 20 μl of MTT was added to each well and incubated for an additional 4 hours before the absorbance was measured at 492/562 nm.

### Transwell assays

To the lower Transwell chambers with or without Matrigel, 800 μl of medium supplemented with 10% FBS was added. For the migration assay, MCF7 cells (5×10^4) were placed into the upper Transwell chambers and incubated for 24 hours. Invasion assay, however, MCF7 cells (10×10^4) were placed into the upper Transwell chambers and incubated for 48 hours. After being stained with crystal violet for 30 minutes, the chambers were examined with an inverted microscope at a magnification of 100× to count the cells that had migrated into the lower chambers. The overall data analysis process is illustrated in **Supplementary Figure S1**.

### Statistical analysis

Statistical evaluations were conducted using R (version 3.6.1). To analyse multiple data groups, we employed analysis of variance (ANOVA), followed by either Student’s t test or the Wilcoxon rank sum test for pairwise comparisons. A p value less than 0.05 was considered statistically significant.

## Results

### Identification of molecular subgroups and TIME evaluation

**Figure 1:**
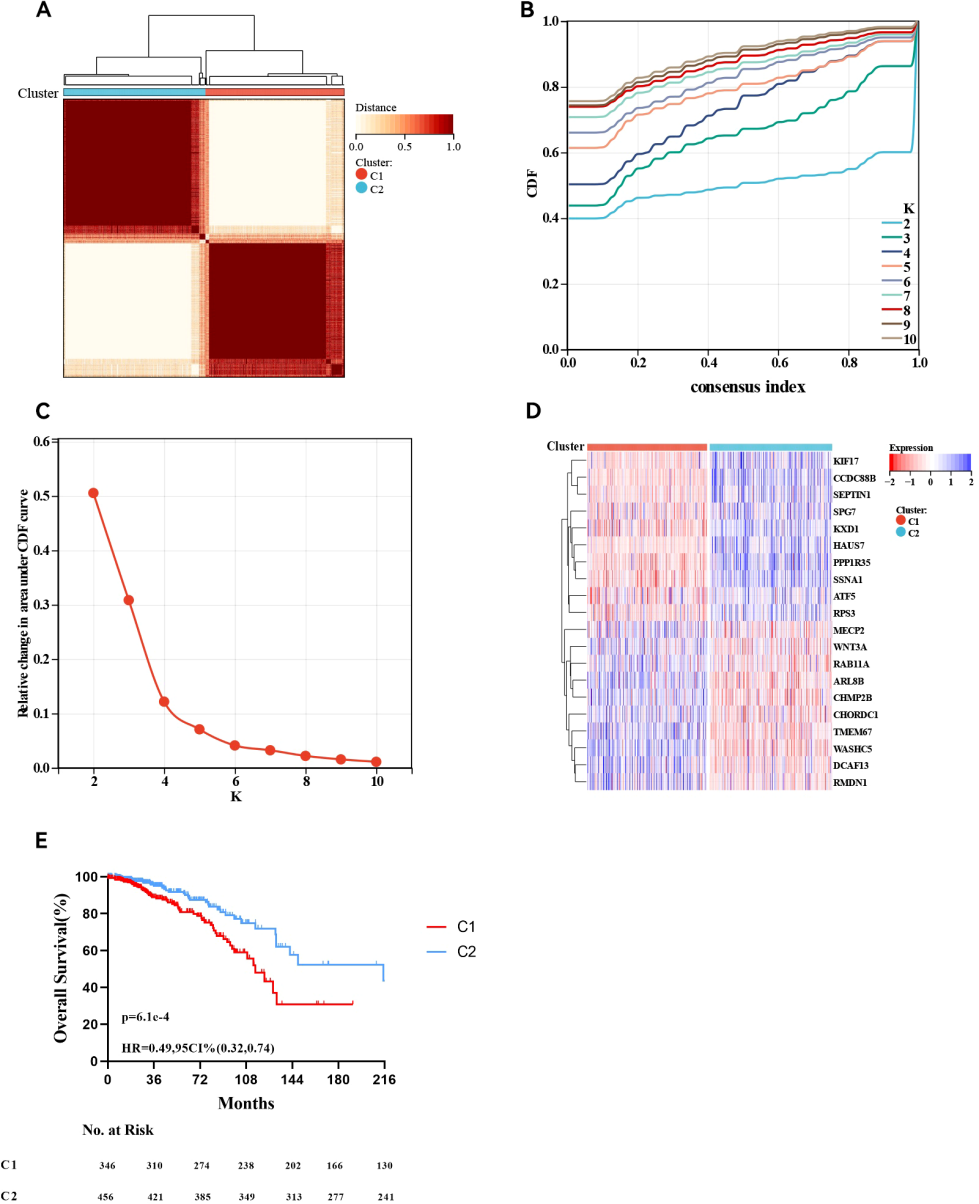
Consensus cluster. **(A–C)** *K* = 2 was identified the optimal value for consensus clustering, **(D)** Heatmap visualising the expression of microtubule-based process-related genes in the two subgroups, **(E)** Survival curve of the patients in the two subgroups.

In total, 803 ER+ breast cancer patients were analysed to identify two molecular subtypes based on MBPRG. Univariate Cox analysis revealed that 120 MBPRGs were strongly associated with OS (P < 0.05, **Supplementary Table S1**). Unsupervised consensus clustering was subsequently utilised to investigate microtubule-based process-related patterns in ER+ breast cancer, guided by the expression profiles of the 120 survival-associated MBPRGs. When K = 2 (**Figures 1A–C**), the optimal cluster stability was assessed from the unsupervised clustering results of the training cohorts. In this analysis, 397 patients were categorised into cluster 1, whereas 406 patients were included in cluster 2. A heatmap illustrated the expression levels of MBPRGs across the two subtypes (**Figure 1D**), revealing significant differences in expression between clusters 1 and 2. Furthermore, patients in cluster 2 demonstrated better overall survival than those in cluster 1 did (P = 6.1e-5, HR = 0.49, 95% CI = 0.32–0.74, **Figure 1E**). These results suggest that MBPRGs successfully classify ER+ breast cancer patients into two molecular subtypes, each of which is associated with distinct overall survival outcomes.

### Patients with these two molecular subtypes present varying TIME and immune profiles

**Figure 2:**
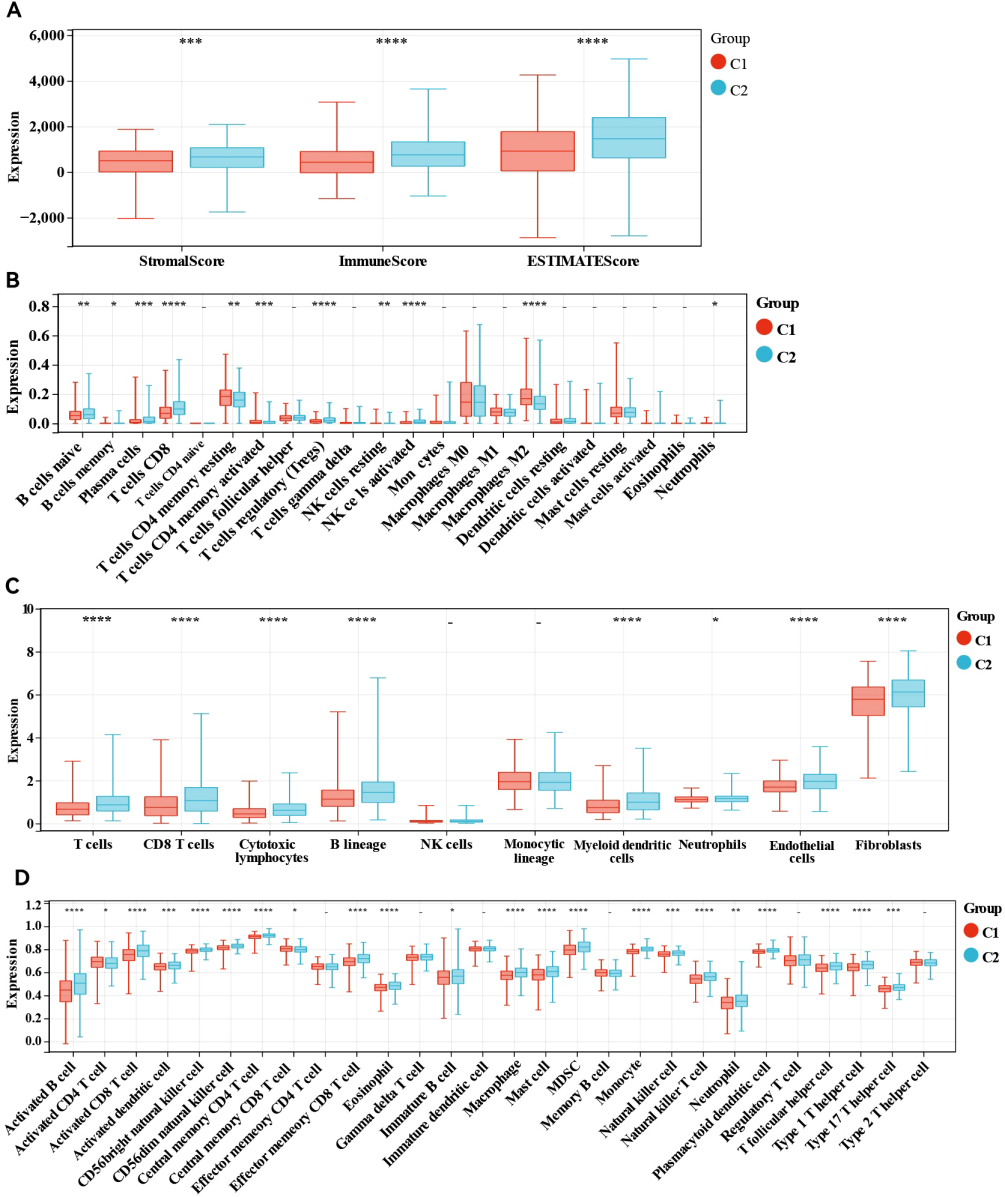
Immune analyses in the two clustered subgroups. **(A)** Stromal score, immune score, ESTIMATE score calculated by ESTIMATE algorithm, **(B)** abundance of tumour immune infiltrating cells evaluated by CIBERSORT algorithm, **(C)** abundance of eight major immune cell types evaluated by MCP algorithm **(D)** abundance of 29 immune related cells evaluated by ssGSEA algorithm. - not available; *p<0.05; **p<0.01; ***p<0.001; ****p<0.0001.

We subsequently conducted immune analyses to investigate the immune variations between the two molecular subtypes. The ESTIMATE algorithm (**Figure 2A**) indicated that ER+ breast cancer patients in cluster 2 had notably higher immune scores (P <0.0001) and ESTIMATE scores (P <0.0001) and higher stromal scores (P <0.001) than those in cluster 1 did. We assessed the differences in the levels of 22 immune cell types between the two clusters using the CIBERSORT algorithm (**Figure 2B**). Patients in cluster 2 presented increased levels of naive B cells, memory B cells, plasma cells, CD8+ T cells, regulatory T (Treg) cells, resting NK cells, activated memory NK cells, and neutrophils, whereas M2 macrophages, resting memory CD4+ T cells, and activated memory CD4+ T cells were less abundant than those in cluster 1. We also examined the differences in the MCP scores between the two groups (**Figure 2C**), employing the MCP-counter algorithm to quantify the absolute abundances of fibroblasts, endothelial cells, and eight different types of immune cells with transcriptomic data. The numbers of cell types that promote immune responses, such as CD8+ T cells, T cells, cytotoxic lymphocytes, B lineage cells, neutrophils and myeloid dendritic cells, were significantly greater in cluster 2, and the absolute abundances of the other two stromal cells, including fibroblasts and endothelial cells, were also significantly greater in cluster 2. Finally, the immune landscape assessed by the ssGSEA algorithm significantly differed between clusters 1 and 2, with cluster 1 exhibiting a relatively low immune status. Statistical analysis revealed that 26 other cell types in cluster 2 were significantly more abundant than those in cluster 1, except for activated CD4 T cells, effector memory CD4 T cells, gamma delta T cells, memory B cells, regulatory T cells, and type 2 T helper cells (**Figure 2D**). These results underscore the notable differences in the TIME and immune profiles between the two molecular subtypes.

### DEG and functional analysis

**Figure 3:**
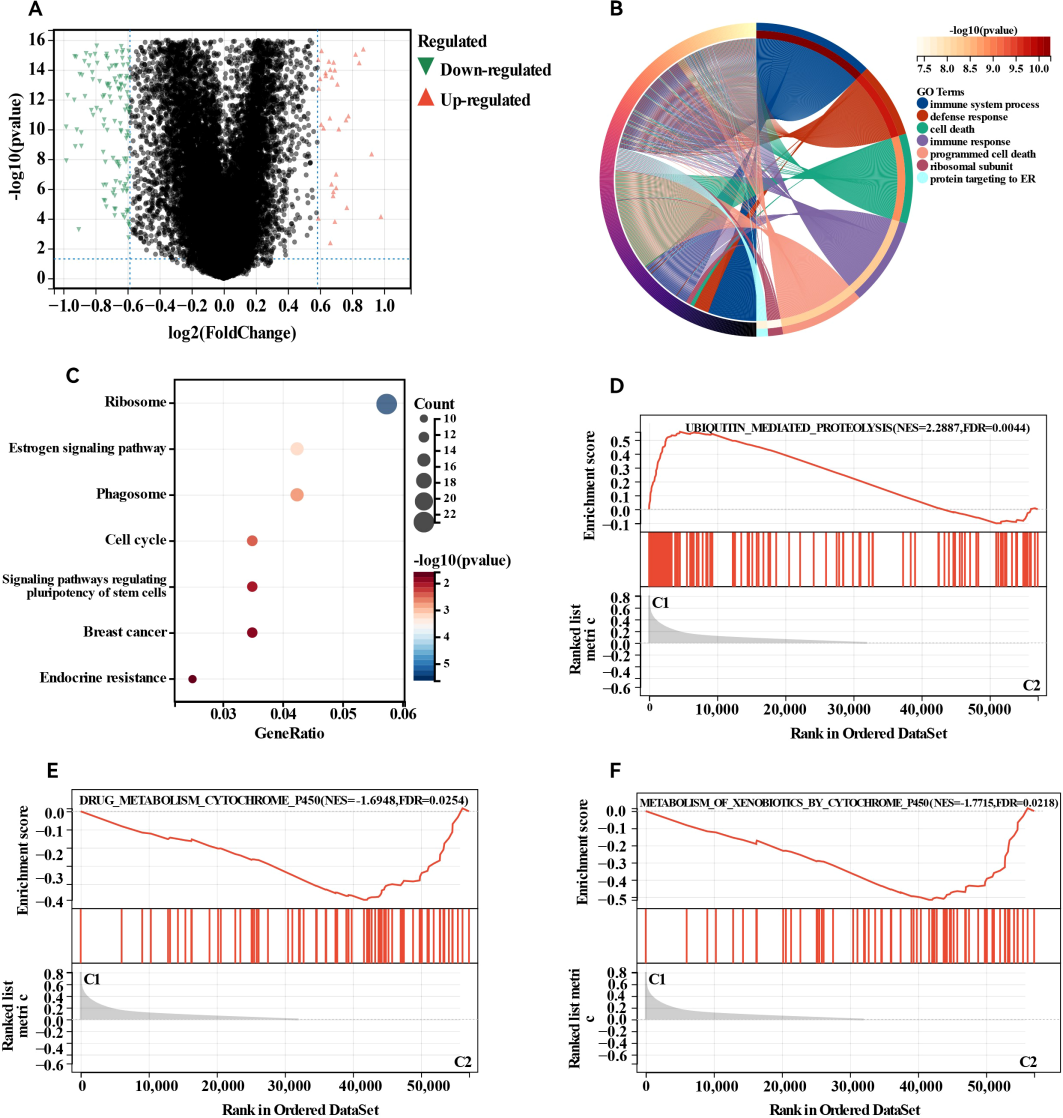
Differentially expressed genes (DEGs) analysis and functional analyses. **(A)** Volcano plot showing the DEGs between the two subgroups, **(B)** circle plot and network visualising the biological processes enriched by gene ontology (GO) analysis, **(C)** bubble diagram showing the signalling pathways enriched by Kyoto Encyclopedia of Genes and Genomes (KEGG) analysis, **(D–F)** GSEA plots visualising the result of GSEA analysis. DEGs, differentially expressed genes; GSEA, gene set enrichment analysis.

We detected DEGs between the two clusters and performed functional analysis to explore potential signalling pathways. A total of 1,026 DEGs were identified, comprising 699 genes whose expression was downregulated and 327 genes whose expression was upregulated in cluster 1 relative to that in cluster 2 (**Figure 3A**). GO enrichment analysis indicated that the overlapping genes influenced biological functions related primarily to immune system processes, defence responses, supramolecular fibre organisation, and the adaptive immune system (**Figure 3B**). Similarly, KEGG enrichment analysis revealed several signalling pathways linked to drug resistance, including the ribosome, phagosome, oestrogen signalling pathway, and endocrine resistance (**Figure 3C**). Additionally, GSEA revealed significant differences in pathways such as ubiquitin-mediated proteolysis, drug metabolism via cytochrome P450, and metabolism of xenobiotics by cytochrome P450 between the two groups (**Figures 3D–F**). Abnormalities in the ubiquitin system may be involved in disease progression by regulating oncoprotein degradation or stress response, while changes in the P450 pathway suggest an imbalance between drug metabolism capacity and xenobiotics detoxification function. Collectively, these results indicate that the expression of MBPRGs is linked to immune responses and drug resistance, which may play a role in the unfavourable prognosis observed in ER+ breast cancer patients.

### Establishment of the MBPRG-based risk model for the training cohort

**Figure 4:**
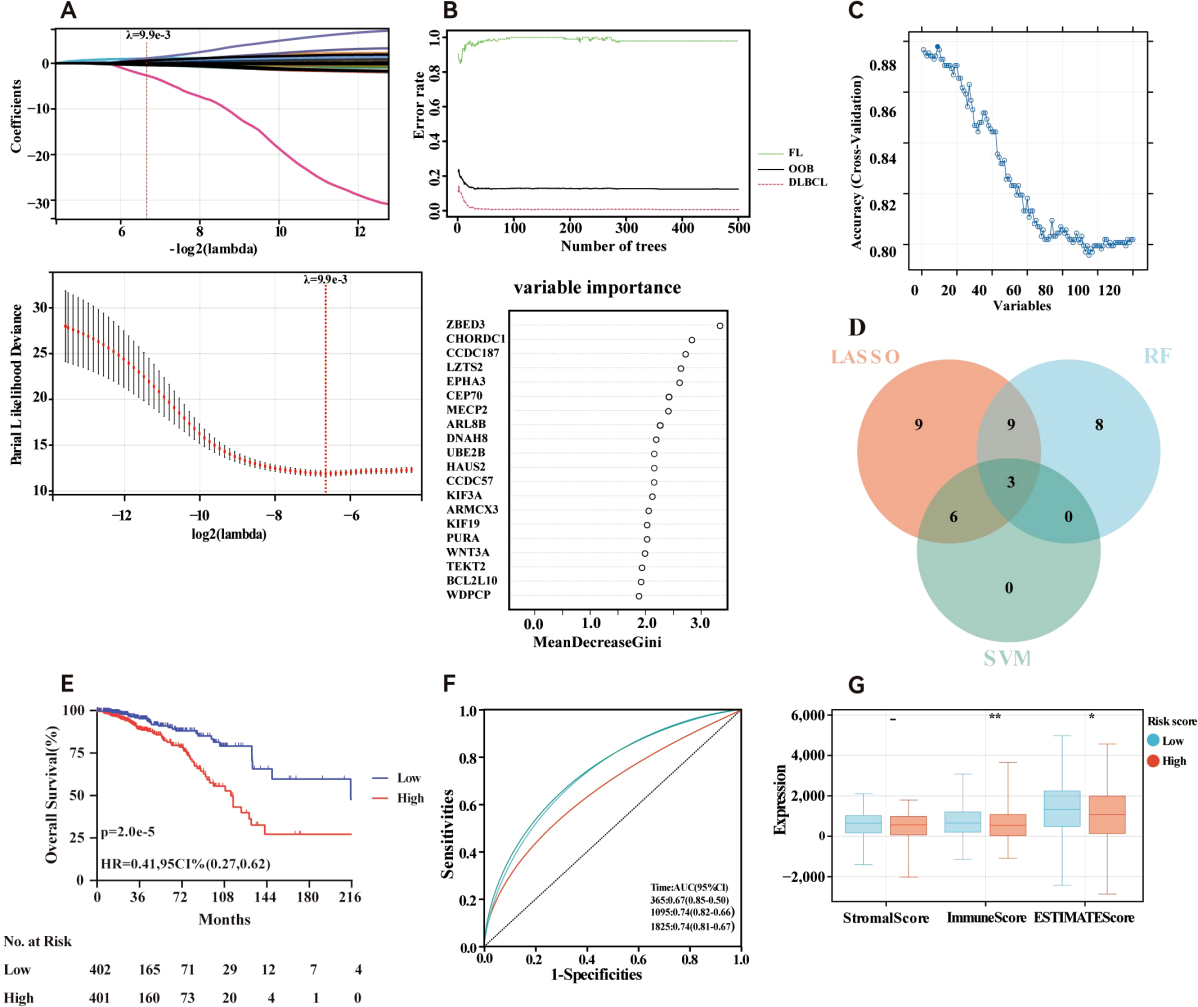
Construction of risk model in the training cohort. **(A)** Fine-tuning the least absolute shrinkage and selection operator (LASSO) model’s feature selection. **(B)** Random forest (RF) for the relationships between the number of trees and error rate and top 20 genes identified in the RF algorithm. **(C)** A plot illustrating the process of selecting biomarkers using the support vector machine (SVM) technique **(D)** Venn diagram showing the central genes identified by machine learning. **(E)** Survival curve of the ER+ breast cancer patients in the two groups. **(F)** Time-dependent ROC curve of the risk model. **(G)** Stomal score, immune score, ESTIMATE score and calculated by ESTIMATE algorithm. - not available; *p<0.05; **p<0.01. ER+, oestrogen receptor-positive.

The risk signature model was designed to assess the prognostic predictive potential of MBPRGs in ER+ breast cancer. LASSO analysis was used to select candidate genes for the risk model, resulting in 37 genes being filtered on the basis of the optimal λ value (**Figure 4A**). The RF algorithm further assessed the significance of these genes, ranking a total of 120 genes. From this ranking, we identified the top twenty most critical genes (**Figure 4B**). Additionally, a machine learning method that employed SVM offered insights into the top nine most significant genes (**Figure 4C**). Using genes identified through LASSO analysis, RF, and SVM, three genes—CHORDC1, WNT3A, and MECP2—were incorporated into a hazard model (**Figure 4D**). This risk model, constructed as outlined in the Methods, effectively categorised ER+ breast cancer patients into high-risk and low-risk categories (P = 2.0e-5, HR = 0.41, 95% CI = 0.27–0.62; **Figure 4E**). To validate the model, ROC curves exhibited strong performance, and time-dependent ROC analysis indicated significant predictive ability over a five-year period, reflected by AUC values of 0.67, 0.74, and 0.74 for one, three, and five years, respectively (**Figure 4F**). Furthermore, the ESTIMATE algorithm was applied to assess the TIME across the two groups, suggesting that the immune score (P <0.01) and ESTIMATE score (P <0.05) were considerably greater in the low-risk group than in the high-risk group. (**Figure 4G**). In conclusion, machine learning-based risk models have strong prognostic potential in ER+ breast cancer patients and are significantly associated with the TIME.

### Independence of the developed risk model

**Figure 5:**
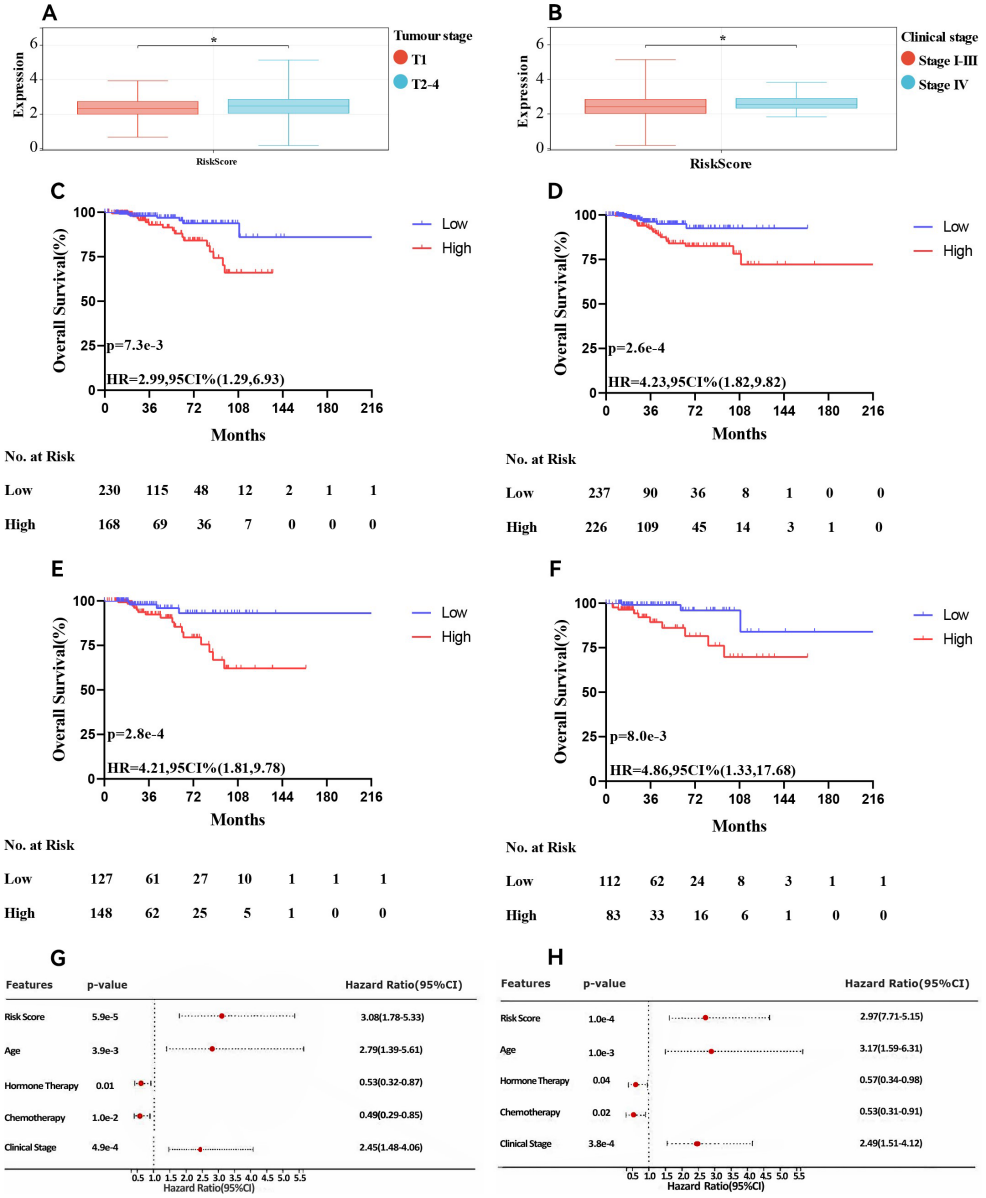
Association of risk score and clinical characteristics. Significant difference was identified in patients with different tumour size **(A)**, clinical stage **(B)**. Survival curve of patients treated with chemotherapy **(C)**, endocrine therapy **(D)**, AI **(E)** and tamoxifen **(F)**. Univariate**(G)** and multivariate **(H)** Cox analyses of clinicopathologic factors and the risk score in ER+ breast cancer patients in the TCGA cohort. * p<0.05. AI, aromatase Inhibitors; ER+, oestrogen receptor-positive; TCGA, The Cancer Genome Atlas.

**Figure 6:**
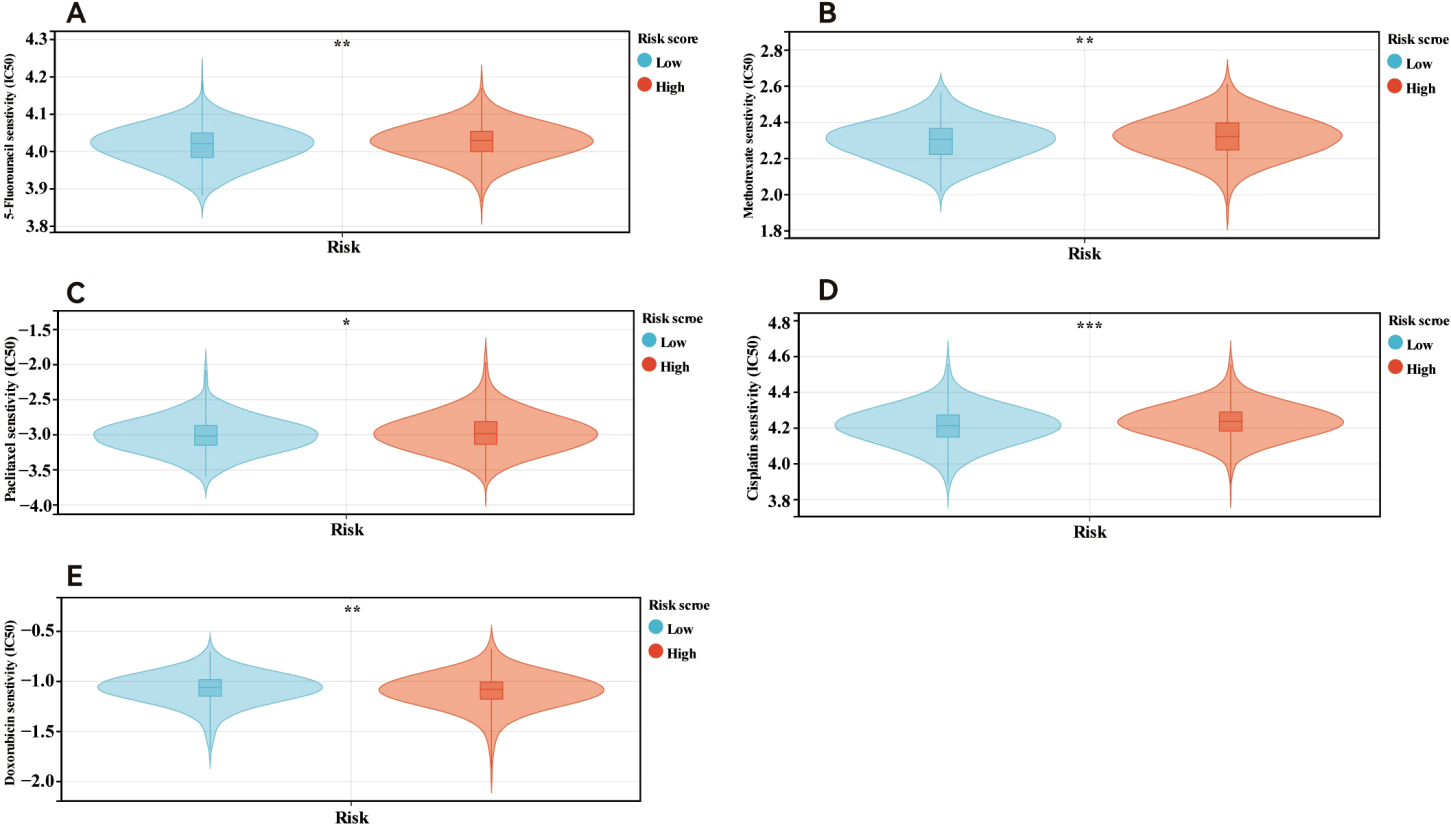
Drug sensitivity in patients in different risk score. Patients within low risk score were more sensitive to 5-fluorouracil **(A)**, methotrexate **(B)**, paclitaxel**(C)** and cisplatin **(D)**. Patients in high risk score were more sensitive to doxorubicin **(E)**. *p<0.05; **p<0.01; ***p<0.001.

Additionally, we examined the relationships between the risk scores and clinical features, evaluating the independence of our risk models via subgroup and regression analyses. When we categorised primary tumour size into cT1 versus cT2-4 (P <0.05, **Figure 5A**) and clinical stage into cStage I-III versus cStage IV (P <0.05, **Figure 5B**). We also analysed how the correlation of the risk score with prognosis varied across various treatment regimens. The risk scores effectively distinguished between ER+ breast cancer patients undergoing chemotherapy (P = 7.5e-3, HR = 2.99, 95% CI = 1.29–6.93, **Figure 5C**) and those receiving endocrine therapy (P = 2.6e-4, HR = 4.23, 95% CI = 1.82–9.82, **Figure 5D**). Further analysis of the endocrine therapy subgroup revealed significant associations between the risk score and prognosis for patients treated with AI (P = 2.8e-4, HR = 4.21, 95% CI = 1.81–9.78, **Figure 5E**) and tamoxifen (P = 8.0e-3, HR = 4.86, 95% CI = 1.33–17.68, **Figure 5F**). Univariate and multivariate Cox regression analyses (**Figures 5G, H**) confirmed that the risk score served as an independent prognostic indicator for patients with ER+ breast cancer, highlighting the robustness of the constructed model. Finally, we examined differences in chemosensitivity among ER+ breast cancer patients across various risk score groups. The results indicated that low-risk patients were particularly sensitive to 5-fluorouracil, paclitaxel, methotrexate, and cisplatin (**Figures 6A–D**), whereas high-risk patients showed heightened sensitivity to doxorubicin (**Figure 6E**). These findings will be instrumental in developing personalised treatment strategies for ER+ breast cancer patients.

### Construction and calibration of an integrated nomogram

**Figure 7:**
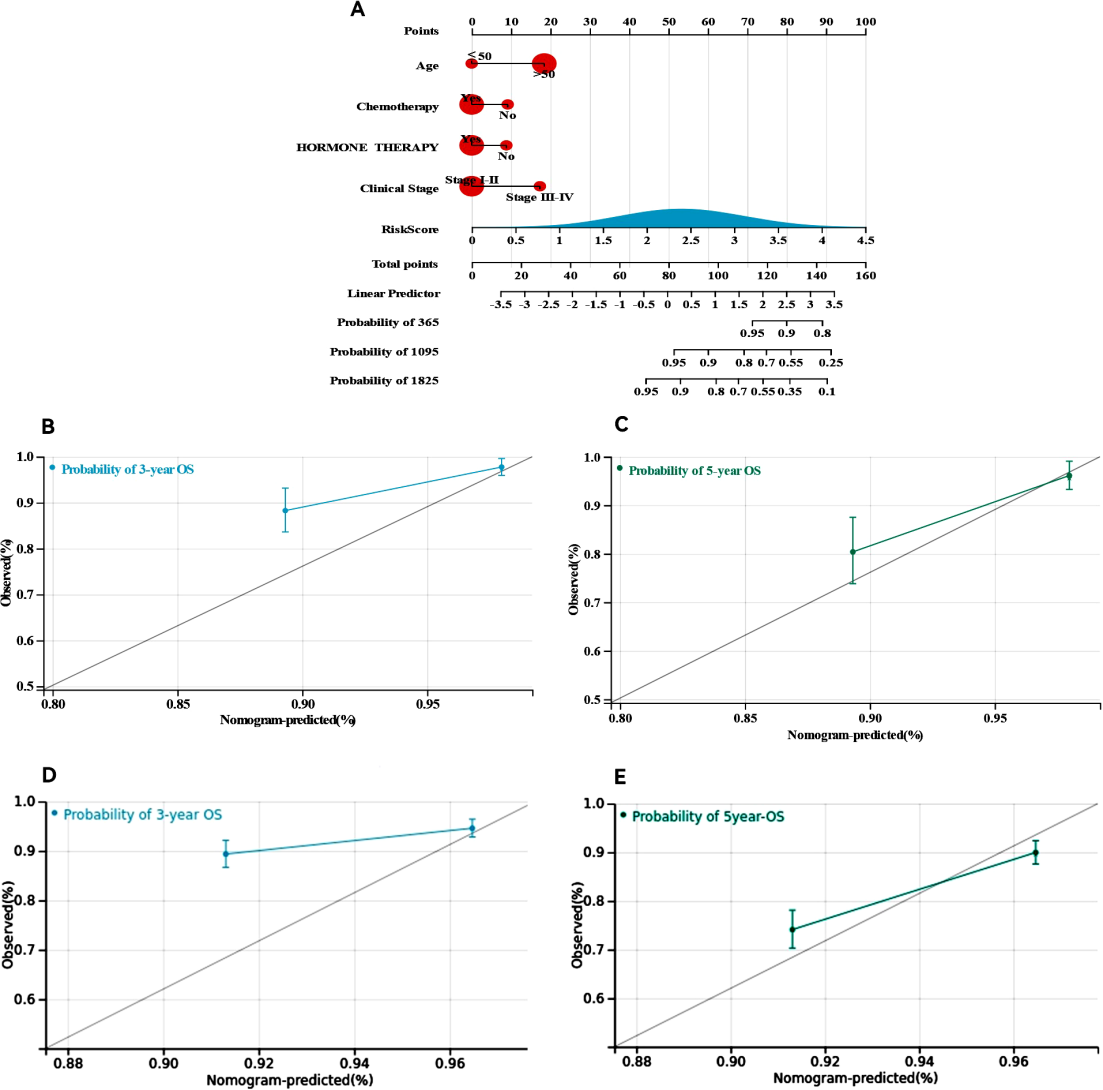
Construction and calibration of nomogram. **(A)** Nomogram integrating risk score and clinical features, **(B, C)** calibration of the nomogram at 3 and 5 years in the training cohort, **(D, E)** calibration of the nomogram at 3 and 5 years in the verification cohort.

We developed nomograms that combine risk models with clinical characteristics to increase the precision of survival prediction for ER+ breast cancer patients, as indicated by the multivariate Cox regression results. The nomograms, illustrated in **Figure 7A**, assign specific scores based on risk factors and pathological features relevant to the prognosis of ER+ breast cancer patients. We then validated these nomograms in both the training and validation cohorts. The diagnostic metrics for the nomogram, including the C-index and calibration curves, indicated satisfactory accuracy, with the C-index for the training cohort reaching 0.7786 (95% CI: 0.7156–0.8416). The observed overall survival closely matched the actual survival rates at 3 and 5 years in the training cohort (**Figures 7B, C**), and similar findings were noted in the validation cohort (**Figures 7D, E**). These findings highlight that comprehensive nomograms can reliably predict the survival of ER+ breast cancer patients.

### ScRNA-seq analysis of key genes

**Figure 8:**
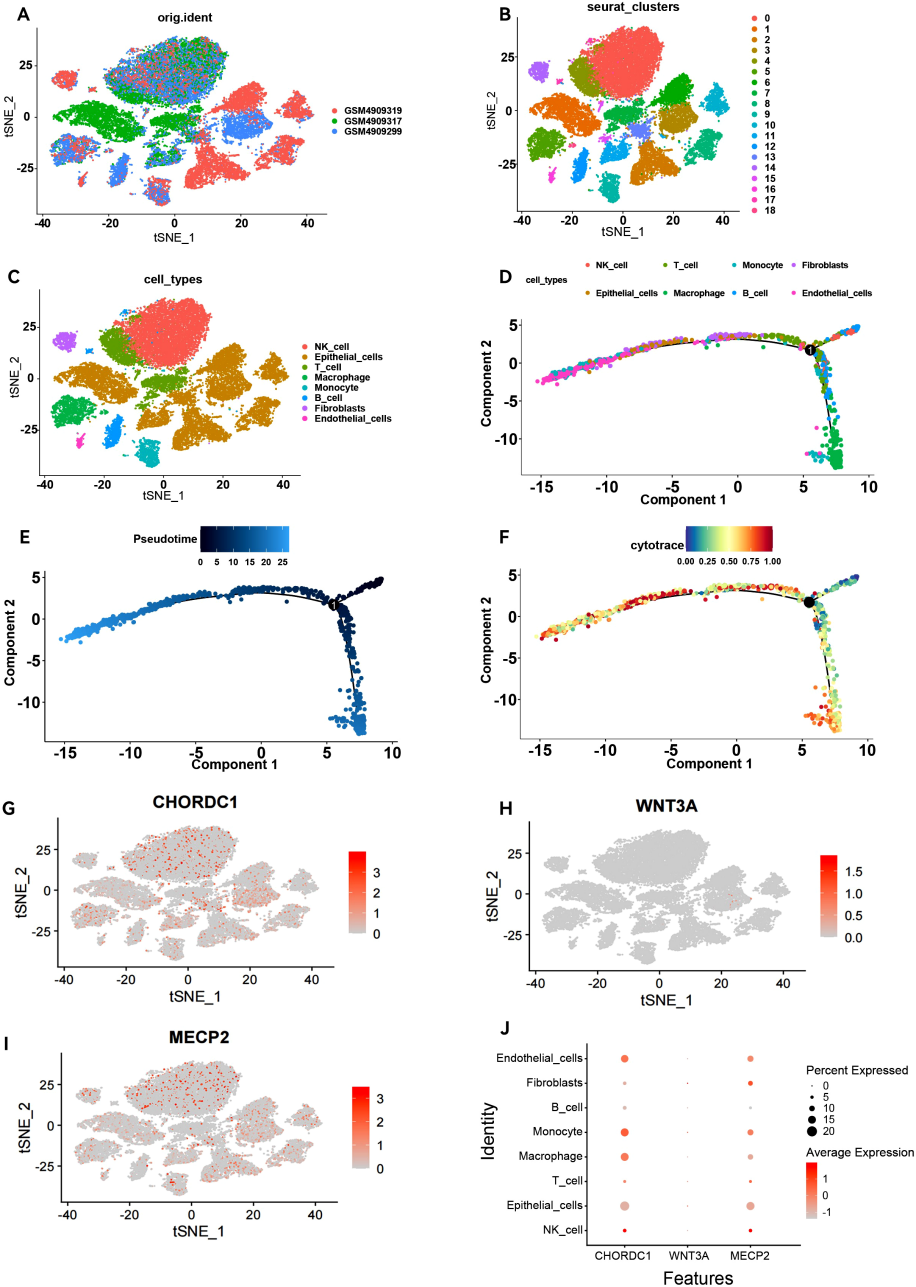
Overview of single-cell atlases in ER positive patients. **(A)** t-SNE clustering plot of 3 samples. **(B)** t-SNE plot depicting clustering of single-cell samples into 18 clusters. **(C)** 8 cell types identified based on marker gene expression. **(D)** Trajectory plot displaying the identified clusters of 8 cell types extracted in scRNA-seq. **(E)** Trajectory plot depicting the developmental time course of 8 cell types. **(F)** UMAP plot displaying the distribution of CytoTRACE score in 8 cell types. T-SNE plot and Dot plot **(J)** highlighting the expression patterns of CHORDC1 **(G)**, WNT3A **(H)** and MECP2 **(I)** for the 8 cell types.

To investigate the differential expression of key genes across various cell types, we performed scRNA-seq analysis on ER+ breast cancer samples. After processing the data, we acquired gene expression profiles from 6,195 cells across three ER+ breast cancer samples for further analysis. We applied PCA to reduce the dimensionality (**Figure 8A**), selected 2,000 variable genes, and identified 18 distinct cell clusters via Seurat (**Figure 8B**). The singleR package facilitated cell type annotation, revealing nine different cell types. Specifically, clusters 5 and 18 were identified as macrophages, cluster 10 as monocytes, and cluster 14 as fibroblasts (**Figure 8C**). Pseudotemporal analysis was used to simulate the developmental paths of various cells on the basis of the expression of temporal genes within single-cell samples. The results revealed a temporal sequence of differentiation, where darker cells transitioned into lighter ones (**Figures 8D, E**), indicating that macrophages and monocytes represent the final stages of differentiation. The CytoTRACE scores vary from 0 to 1, where higher values signify greater stemness (lower levels of differentiation) and vice versa. Additionally, the CytoTRACE score exhibited significant heterogeneity among tumour cells (**Figure 8F**). We determined the expression profiles of three key genes in various cell types: CHORDC1 was expressed predominantly in macrophages, monocytes, and endothelial cells; MECP2 was expressed primarily in macrophages, monocytes, and fibroblasts; WNT3A was lower than CHORDC1 and MECP2 at the overall single-cell level, and its predominant expression was observed in fibroblasts and monocytes. (**Figures 8G–J**).

### Validation of differentially expressed MBPRGs in clinical samples and cell models

**Figure 9:**
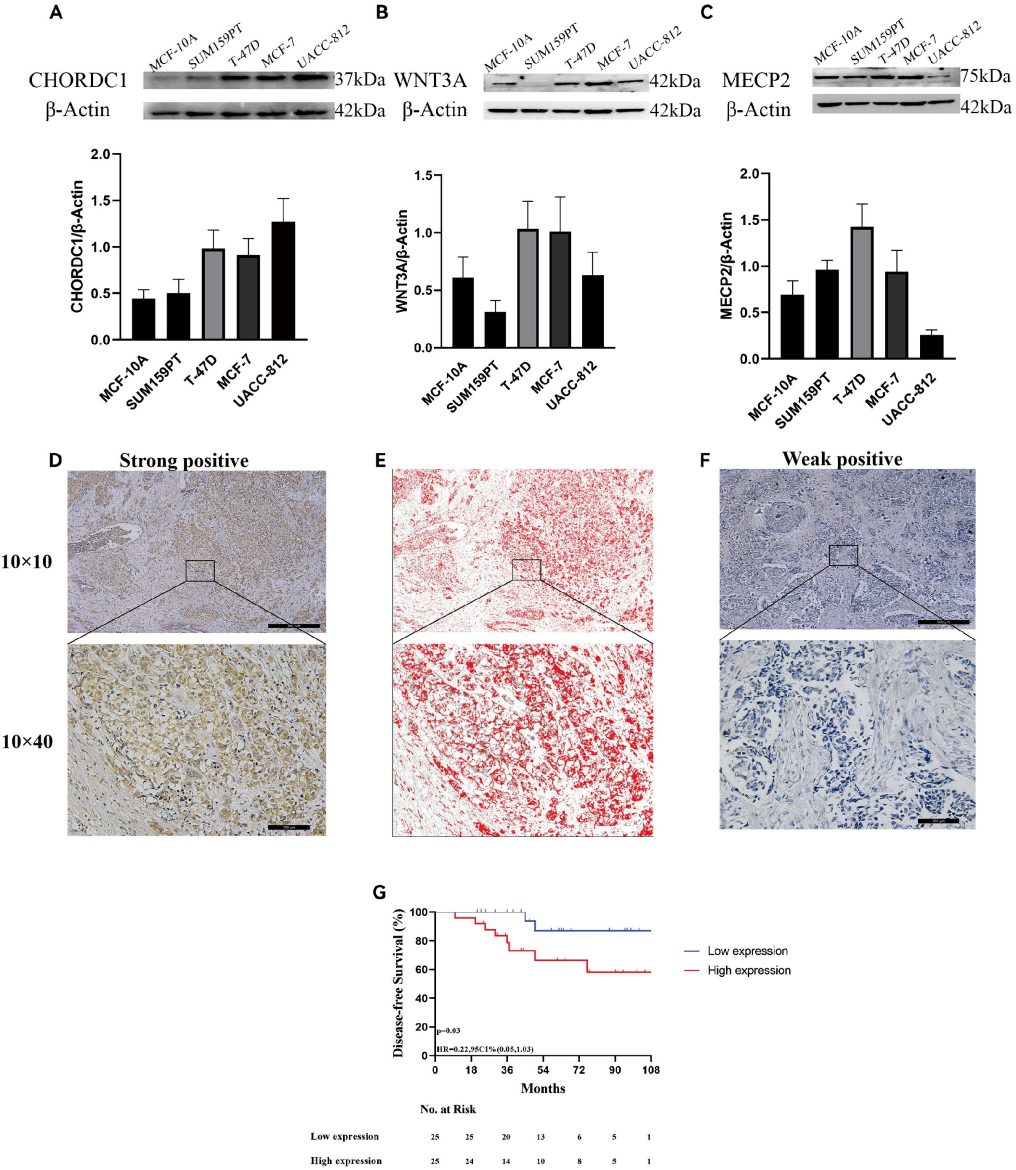
Expression of microtubule-based processes-related genes and associated with ER + breast cancer prognosis. The protein expression of CHORDC1**(A)**, WNT3A **(B)**, MECP2 **(C)** in 5 cell lines and determined by densitometry of protein bands. **(D)** Immunohistochemical (IHC) staining with Diaminobenzidine (DAB) demonstrates strong positivity for the target antigen, indicating high CHORDC1 protein expression levels in ER + breast cancer. **(E)** Image processed by Image-Pro Plus version software. **(F)** IHC staining with DAB demonstrates weak positivity for the target antigen, indicating low expression of CHORDC1 in ER + breast cancer. **(G)** DFS in Harbin Medical University Cancer Hospital cohorts differentiated based on CHORDC1 expression. ER +, oestrogen receptor-positive; DFS, Disease-Free Survival.

To further assess the credibility of the prognostic models related to microtubule-based processes, we first measured the protein levels of three hub genes via Western blotting in various breast cancer cell lines alongside the normal breast epithelial cell line MCF-10A. CHORDC1 was highly expressed in T-47D, MCF-7, and UACC-812 cells but expressed at low levels in MCF-10A and SUM159-PT cells (**Figure 9A**). MECP2 was highly expressed in SUM159-PT, T-47D, and MCF-7 cells but was expressed at lower levels in MCF-10A and UACC-812 cells (**Figure 9B**). WNT3A was predominantly expressed in T-47D and MCF-7 cells, with lower levels in MCF-10A, SUM159-PT, and UACC-812 cells (**Figure 9C**).

Since MECP2 and WNT3A have been well researched in breast cancer, we sought to explore CHORDC1 expression in ER+ breast cancer patients receiving endocrine therapy and chemotherapy. We randomly selected 50 early-stage ER+ breast cancer patients from Harbin Medical University Cancer Hospital for immunohistochemical analysis. The patients’ ages varied from 32 to 75 years, with a median age of 50 years, with pTNM stages I, II, and III comprising 9 (18.0%), 30 (60.0%), and 11 (22.0%) patients, respectively. Among these patients, 12 (24.0%) were HER2 positive, and 38 (76.0%) were HER2 negative; additionally, 21 (42.0%) had Ki-67 levels above 20%, while 29 (48.0%) had levels below 20% (**Table 1**). CHORDC1 expression was primarily localised in the cytoplasm of BC cells (**Figures 9D–F**). The results were measured by assessing the positive area in comparison with the total area, and patients were divided into groups with high and low expression levels. The baseline characteristics of both groups are detailed in **Table 1**, with chi-square analysis indicating a significant correlation between CHORDC1 expression and Ki-67 levels in ER+ breast cancer patients (P = 0.02). After a median follow-up period of 49 months, the 4-year DFS rates were 85.0% for the low-expression group and 64.8% for the high-expression group (P = 0.03, HR = 0.22, 95% CI = 0.05–1.03, **Figure 9G**). Nonetheless, there was no statistically significant difference in the 4-year OS between the two groups (94.0% vs. 92.9%, P = 0.98, **Supplementary Figure S2**).

**Table 1:** Chi-square test analysis of the connection between CHORDC1 expression and clinicopathological features in HMU Cancer Hospital cohort.

Characteristics	High expression (N=25)	Low expression (N=25)	Total (N=50)	P value
Age (years)				1.00
≤50	12 (24.00%)	12 (24.00%)	24 (48.00%)	
>50	13 (26.00%)	13 (26.00%)	26 (52.00%)	
Tumour size classification				0.59
T1	11 (22.00%)	12 (24.00%)	23 (46.00%)	
T2	13 (26.00%)	13 (26.00%)	26 (52.00%)	
T3	1 (2.00%)	0 (0%)	1 (2.00%)	
Lymph node classification				0.73
N0	9 (18.00%)	7 (14.00%)	16 (32.00%)	
N1	11 (22.00%)	12 (24.00%)	23 (46.00%)	
N2	5 (10.00%)	5 (10.00%)	10 (20.00%)	
N3	0 (0%)	1 (2.00%)	1 (2.00%)	
pTNM stage				0.54
I	6 (12.00%)	3 (6.00%)	9 (18.00%)	
II	14 (28.00%)	16 (32.00%)	30 (60.00%)	
III	5 (10.00%)	6 (12.00%)	11 (22.00%)	
Adjuvant chemotherapy				1.00
No	2 (4.00%)	3 (6.00%)	5 (10.00%)	
Yes	23 (46.00%)	22 (44.00%)	45 (90.00%)	
Endocrine therapy				0.34
AI	16 (32.00%)	20 (40.00%)	36 (72.00%)	
Tamoxifen	9 (18.00%)	5 (10.00%)	14 (28.00%)	
HER2 status				0.32
Negative	21 (42.00%)	17 (34.00%)	38 (76.00%)	
Positive	4 (8.00%)	8 (16.00%)	12 (24.00%)	
Ki67				0.02
≤20	10 (20.00%)	19 (38.00%)	29 (58.00%)	
>20	15 (30.00%)	6 (12.00%)	21 (42.00%)	
Surgery type of breast				1.00
Partial mastectomy	2 (4.00%) 23 (46.00%)	2 (4.00%) 23 (46.00%)	4 (8.00%) 46 (92.00%)	
Surgery type of axilla				0.74
ALND	18 (36.00%)	20 (40.00%)	38 (76.00%)	
SLNB	7 (14.00%)	5 (10.00%)	12 (24.00%)	

HER-2, human epidermal growth factor receptor 2; SLNB, sentinel lymph node biopsy; ALND, axillary lymph node dissection.

### CHORDC1 restrains cell viability and invasion and decreases drug sensitivity to tamoxifen and paclitaxel *in vitro*

**Figure 10:**
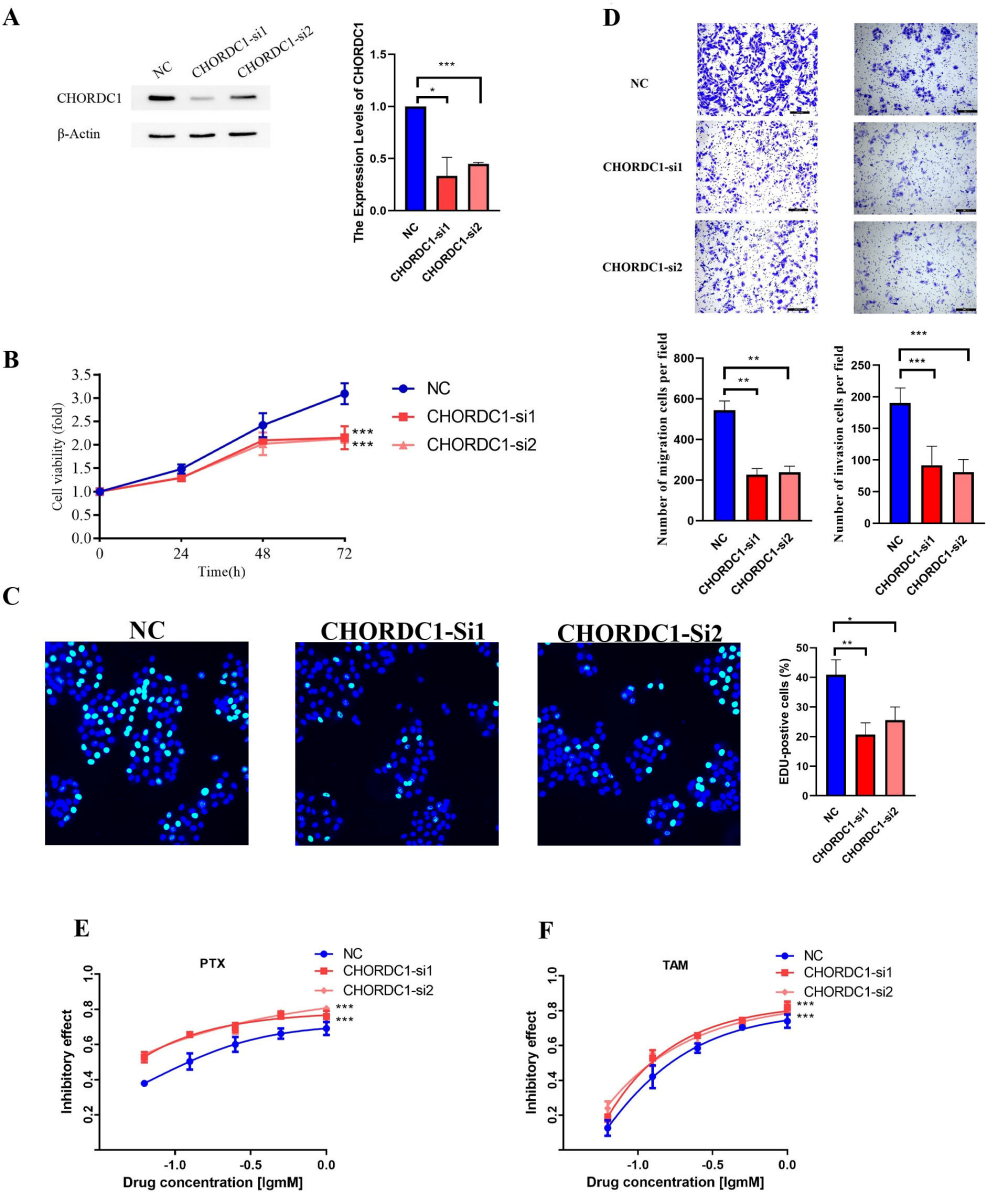
Silencing CHORDC1 suppressed cell viability and invasion and improved drug sensitivity *in vitro*. **(A)** The siRNA effects (NC, si-1 and si-2) were examined by western blot assays. The relative proliferation rates of MCF-7 cells were measured by MTT **(B)** and EDU **(C)** assays in cells with the NC or transiently silenced CHORDC1 (si-1 and si-2). **(D)** Invasive abilities were detected with Transwell assays with or without Matrigel, in cells with the NC or CHORDC1 transiently silenced (si-1 and si-2). Drug sensitivity of paclitaxel **(E)** and tamoxifen **(F)** were detected with MTT assays in cells with the NC or CHORDC1 transiently silenced (si-1 and si-2). *p<0.05; **p<0.01; ***p<0.001. NC, negative control.

In the MCF-7 cell line, CHORDC1 expression was promptly reduced by siRNA transfection (**Figure 10A**). MTT and EDU assays revealed that the viability of ER+ breast cancer cells with CHORDC1 knockdown was lower than that of the corresponding negative control cells (**Figures 10B, C**). Transwell assays revealed that decreased CHORDC1 expression could significantly hinder the invasion and migration of breast cancer cells (**Figure 10D**). Additionally, we investigated the function of CHORDC1 in relation to drug resistance; MCF-7 cells treated with siRNA directed against CHORDC1 presented a significant increase in sensitivity to paclitaxel (**Figure 10E**) and tamoxifen (**Figure 10F**). These findings suggest that CHORDC1 may promote the progression of ER+ breast cancer cells and is crucial for restoring their sensitivity to chemotherapy.

## Discussion

Current recommendations from the American Society of Clinical Oncology suggest that tests should be deemed positive if a minimum of 1% of tumour nuclei show staining for markers within the context of appropriate internal and external controls (27). While new criteria have been established for categorising breast cancer, not all tumours expressing ERs are genuinely responsive to the ER pathway. Furthermore, although some patients may initially respond to treatment, they often develop secondary or “acquired” resistance (28). In early-stage cases, chemotherapy is selected based on the stage and biology of the tumour and is utilised after endocrine resistance is encountered in advanced stages. Even as new therapies, including innovative endocrine agents and antibody–drug conjugates, reshape treatment approaches, significant heterogeneity among ER+ breast cancer patients means that patients continue to face drug resistance (29).

In recent years, taxanes have been commonly employed as microtubule-targeting agents in adjuvant and neoadjuvant treatments for breast cancer (30, 31). Additionally, the effectiveness and safety of nontaxane microtubule polymerisation inhibitors have been increasingly studied in this context (32). Research has also demonstrated that certain pro-oncogenes can influence drug sensitivity through their effects on microtubule-related processes (33), highlighting the potential of microtubules as therapeutic targets. In addition, high expression of microtubule-associated genes is associated with poor clinical outcomes in cancer patients, including reduced overall survival and recurrence-free survival, suggesting that these genes may serve as prognostic biomarkers (12, 34). Considering these elements, we established a comprehensive evaluation system using bioinformatics to identify hub genes and molecular pathways linked to drug resistance in ER+ breast cancer that are connected to microtubule processes. Our aim was to improve the understanding of the physiological and molecular mechanisms that lead to prognosis in patients with ER+ breast cancer and to provide insights into prognostic biomarkers and therapeutic targets.

Consensus clustering serves as an effective approach for categorising samples into distinct subcategories on the basis of gene expression information. By utilising the expression matrix of MBPRGs in patients with ER+ breast cancer, we initially identified two molecular subgroups whose overall survival rates significantly differed. Next, we conducted immunological and functional analyses to assess the impact of the TIME on ER+ breast cancer. As previously noted, the TIME is crucial for patient prognosis, considering that tumour development is associated with alterations in the surrounding stroma, where immune cells play crucial roles in the tumour microenvironment (35). To characterise the TIMEs within both subgroups, we utilised ESTIMATE, CIBERSORT, and MCP analyses. Our results suggest that patients with a better prognosis have elevated immune scores and a more positive immune landscape, reflecting differences in the abundance of immune cells. Functional analyses of the two subgroups were conducted to investigate the possible biological mechanisms involved. Both the GO and KEGG analyses of the identified DEGs suggested that immune responses and drug resistance might facilitate the impact of microtubule-related processes on the onset and progression of ER+ breast cancer.

In recent years, with the rapid advancement of bioinformatics technology, machine learning has emerged as a pivotal approach for screening prognostic markers, accompanied by an exponential growth in related literature (36). In the context of modelling, machine learning allows algorithms to derive insights from errors, process datasets, identify patterns, and generate informed predictions with limited human intervention. The field of machine learning is typically categorised into supervised learning, unsupervised learning, semi-supervised learning, and reinforcement learning (37). In the context of machine learning modelling, stochastic result aggregations are highly contingent upon data partitioning strategies and random seed initialisation, thereby compromising the reproducibility of identified markers. Machine learning methodologies enhance model robustness via techniques such as cross-validation and stability selection. As an illustration, in microRNA biomarker screening for breast cancer, the feature subset derived by SVM-based recursive feature elimination demonstrated superior Kuncheva index values and percentages of overlapping genes/features compared to differential expression analysis results across both TCGA and GEO datasets. This approach yielded a 20%+ improvement in classification performance metrics (38). Ensemble learning strategies significantly mitigate model variance and bias by integrating multiple base learners. For instance, in osteosarcoma prognostic research, investigators combined CoxBoost with gradient boosting machines to develop the AIDPI model, which exhibited a significantly higher mean C-index than single-algorithm approaches in predicting patient survival probabilities (39).

By leveraging the advantages of machine learning approaches and further exploring the prognostic significance of MBPRGs in patients with ER+ breast cancer, we identified MBPRGs associated with prognosis by using three machine learning algorithms to identify key genes and develop a prognostic risk model. Specifically, LASSO identified 27 candidate genes, whereas SVM and RF selected 9 and 20 genes, respectively. Through cross-validation across the three algorithms, three hub genes—CHORDC1, WNT3A, and MECP2—were unanimously identified across the different modelling approaches. These advantages manifest in two key aspects. Firstly, genes consistent across multiple algorithms are preferentially retained, thereby mitigating biases inherent to individual modelling approaches. Secondly, individual machine learning algorithms often face theoretical limitations—for example, logistic regression struggles to model non-linear relationships, while decision trees are prone to overfitting. By integrating diverse algorithms, the combined approach substantially enhances the predictive performance and biological relevance of prognostic models through algorithmic complementarity, feature stabilisation, and comprehensive data integration.

Additionally, these curves demonstrated excellent discriminatory power in the validation dataset. We also created a nomogram that incorporates the risk score, age, chemotherapy and endocrine therapy status, and clinical stage to provide direct survival predictions for ER+ breast cancer patients. The prognostic relevance of CHORDC1 was subsequently validated in our cohort and through *in vitro* studies. In conclusion, the risk model derived from the three hub genes may offer valuable insights and recommendations for clinical treatment decisions.

To summarise, our research developed and confirmed a prognostic model related to microtubule processing that incorporates CHORDC1, WNT3A, and MeCP2. We demonstrated that these factors may affect drug resistance in ER+ breast cancer by influencing immune responses and immune cell behaviour. Additionally, we found that CHORDC1 levels are significantly associated with the outcomes of patients undergoing chemotherapy and endocrine therapy. Reducing CHORDC1 expression leads to decreased cell viability and invasiveness, as well as diminished sensitivity to tamoxifen and paclitaxel *in vitro*.

Therefore, our findings suggest potential targets for enhancing the effectiveness of drug therapies. However, our study has certain limitations. First, further validation with a larger population is needed to confirm the reliability of the microtubule-related prognostic model. Second, the development and validation of the prognostic model relied on retrospective public data, necessitating clinical trials to ensure its reliability and applicability. Moreover, both *in vitro* and *in vivo* studies are needed to explore the fundamental mechanisms of these essential genes in ER+ breast cancer.

## Conclusions

In conclusion, our prediction model, which is based on 3 MBPRGs and the clinical characteristics of patients, can reliably predict the drug response of patients with ER+ breast cancer. These 3 MBPRGs thus appear to play important roles in the development and progression of ER+ breast cancer.

## Data Availability

The original contributions presented in the study are included in the article/**Supplementary Material**. Further inquiries can be directed to the corresponding authors.
