# Supplementary material for: Association of microtubule-based processes gene expression with immune microenvironment and its predictive value for drug response in oestrogen receptor-positive breast cancer
Source: Front Immunol. 2025 Jul 30;16:1608991. doi: 10.3389/fimmu.2025.1608991 (PMC12343727; doi:10.3389/fimmu.2025.1608991)
Supplement: Supplementary file 1 [file DataSheet1.doc]

Figure S1The flowchart of this study.


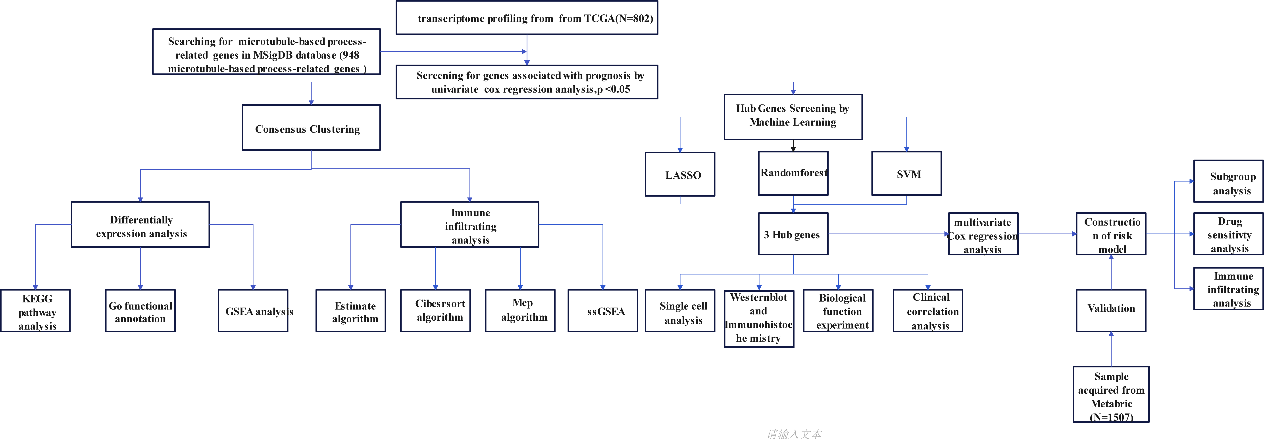


Figure S2 OS in Harbin Medical University Cancer Hospital cohorts diûerentiated based on CHORDC1 expression.


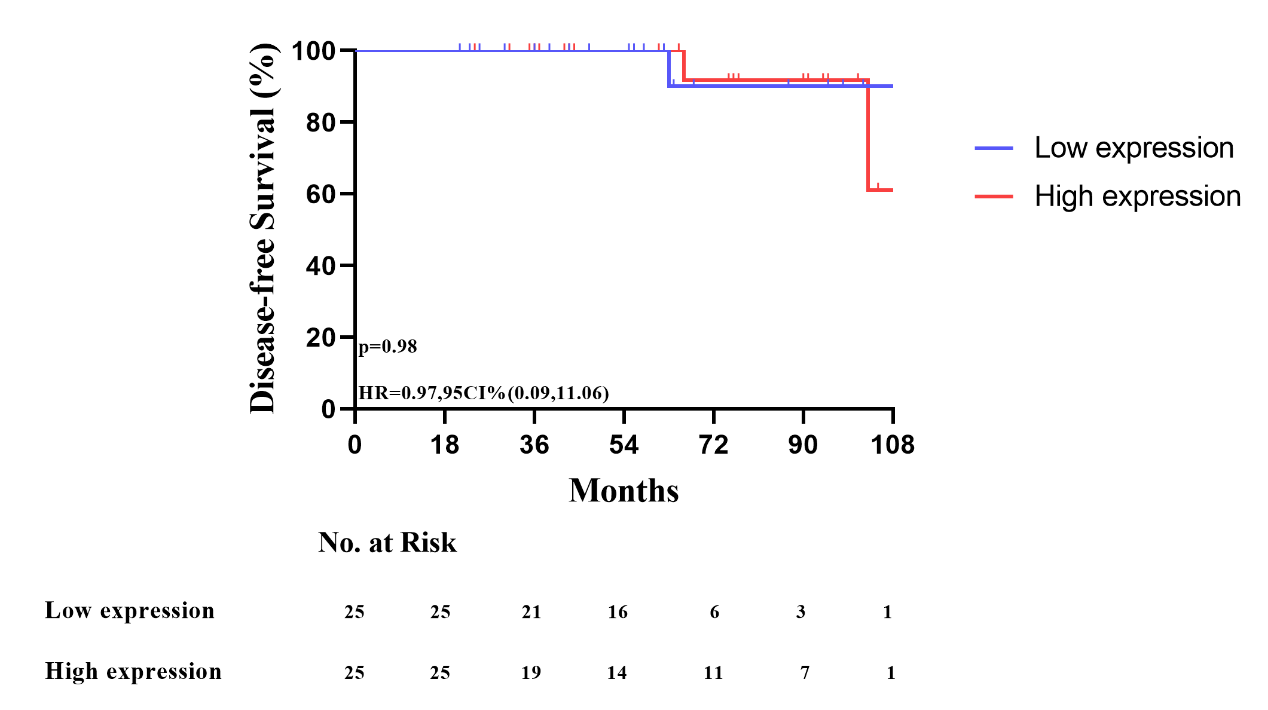


Table S1. Microtubule-based processes -related genes associated with ER + BC prognosis.

| Tag | HR | Lower | Upper | Likelihood | logrank | Wald |
| --- | --- | --- | --- | --- | --- | --- |
| WNT3A | 2.35E+30 | 5.78E+17 | 9.57E+42 | 0.00114 | 4.74E-88 | 2.35E-06 |
| VBP1 | 6.02E+08 | 136366.7 | 2.66E+12 | 0.00114 | 4.74E-88 | 2.35E-06 |
| RPS3 | 2.15E+12 | 11820523 | 3.93E+17 | 0.004957 | 2.71E-29 | 4.33E-06 |
| KIF3A | 18.75977 | 5.408898 | 65.06482 | 0.003688 | 1.9E-25 | 3.83E-06 |
| UBXN2B | 6.4E+08 | 74038.44 | 5.53E+12 | 0.007054 | 4.62E-22 | 1.16E-05 |
| APC | 3897.529 | 113.2939 | 134082.6 | 0.002366 | 9.94E-18 | 4.65E-06 |
| WASHC5 | 2.18E+10 | 2920815 | 1.63E+14 | 0.00068 | 3.23E-17 | 1.68E-07 |
| FBXW11 | 51.07477 | 9.53219 | 273.6655 | 0.004598 | 3.68E-17 | 4.38E-06 |
| AP3M1 | 1.51E+36 | 1.86E+20 | 1.22E+52 | 0.005327 | 9.53E-17 | 8.3E-06 |
| TMEM67 | 24.35451 | 5.796575 | 102.3263 | 0.006307 | 1.77E-16 | 1.3E-05 |
| PPP1R35 | 3.679195 | 1.977722 | 6.844477 | 0.010422 | 4.44E-16 | 3.9E-05 |
| KIF5B | 41801.69 | 635.3843 | 2750118 | 0.001325 | 5.02E-16 | 6.31E-07 |
| STAU2 | 5.895529 | 2.529326 | 13.74171 | 0.01049 | 5.03E-16 | 3.97E-05 |
| CHMP4A | 702949.4 | 2466.318 | 2E+08 | 0.002594 | 2.31E-15 | 3.04E-06 |
| CHORDC1 | 1.6E+108 | 4.23E+55 | 6E+160 | 0.011605 | 8.95E-15 | 5.49E-05 |
| SENP6 | 35.64473 | 10.60914 | 119.7597 | 0.000126 | 7.66E-14 | 7.49E-09 |
| ARMC12 | 1.41E+40 | 1.87E+20 | 1.07E+60 | 0.012808 | 1.19E-13 | 7.53E-05 |
| PAFAH1B1 | 298681.4 | 1822.7 | 48944186 | 0.002386 | 1.34E-13 | 1.26E-06 |
| DCAF13 | 1.662996 | 1.324203 | 2.088467 | 0.005839 | 2.09E-13 | 1.21E-05 |
| EPHA3 | 6.08E+41 | 2.42E+24 | 1.53E+59 | 0.002389 | 7.28E-13 | 2.52E-06 |
| KIF3B | 247474.6 | 488.7497 | 1.25E+08 | 0.013449 | 9.27E-13 | 9.28E-05 |
| EML3 | 2.864266 | 1.91558 | 4.282786 | 0.00081 | 1.23E-12 | 2.95E-07 |
| EZR | 10.8008 | 3.376169 | 34.55316 | 0.011285 | 2.61E-12 | 6.05E-05 |
| HAUS2 | 9.110895 | 3.605622 | 23.02194 | 0.001749 | 4.43E-12 | 2.99E-06 |
| TTC21B | 5.937104 | 2.657424 | 13.26442 | 0.005072 | 5.91E-12 | 1.41E-05 |
| CCDC57 | 16.41426 | 4.11489 | 65.47634 | 0.011475 | 6.15E-12 | 7.37E-05 |
| CCDC88B | 24160.91 | 93.23647 | 6260959 | 0.024385 | 6.84E-12 | 0.000372 |
| FES | 12.16601 | 3.562154 | 41.55123 | 0.010726 | 1.74E-11 | 6.69E-05 |
| ARMCX3 | 2083141 | 1050.143 | 4.13E+09 | 0.01661 | 4.24E-11 | 0.000173 |
| ARL8B | 8.363181 | 3.383399 | 20.67235 | 0.001933 | 5.36E-11 | 4.23E-06 |
| DEFB1 | 32.19953 | 6.478201 | 160.0459 | 0.005685 | 6.45E-11 | 2.2E-05 |
| HSPA8 | 1.682563 | 1.315023 | 2.152827 | 0.007889 | 1.67E-10 | 3.51E-05 |
| RAB1A | 8.07E+11 | 1900577 | 3.42E+17 | 0.008372 | 2.02E-10 | 3.37E-05 |
| CEP131 | 1.41E+46 | 3.31E+25 | 6E+66 | 0.003549 | 2.35E-10 | 1.16E-05 |
| KIFBP | 46.21925 | 6.393513 | 334.1229 | 0.015913 | 2.5E-10 | 0.000146 |
| KIF19 | 4.28E+20 | 2.23E+12 | 8.23E+28 | 0.000999 | 3.01E-10 | 1.05E-06 |
| KLC1 | 3.163193 | 2.111391 | 4.738958 | 1.29E-05 | 5.05E-10 | 2.36E-08 |
| ATRX | 312.7538 | 32.13788 | 3043.602 | 0.001269 | 5.31E-10 | 7.46E-07 |
| XRCC3 | 120.6571 | 11.42749 | 1273.958 | 0.010327 | 7.3E-10 | 6.73E-05 |
| PHLDB2 | 415.5505 | 16.0199 | 10779.23 | 0.018946 | 5.89E-09 | 0.000284 |
| DNAH8 | 1633.144 | 32.88803 | 81098.21 | 0.0175 | 6.88E-09 | 0.000205 |
| CRYAB | 1.12E+09 | 18531.16 | 6.82E+13 | 0.017541 | 9.44E-09 | 0.000208 |
| WDR35 | 1522.665 | 60.16532 | 38535.65 | 0.002038 | 1.03E-08 | 8.78E-06 |
| STAG2 | 106.2992 | 11.09468 | 1018.463 | 0.007454 | 1.47E-08 | 5.18E-05 |
| TAOK1 | 2240082 | 3166.83 | 1.58E+09 | 0.005537 | 1.62E-08 | 1.26E-05 |
| LMNA | 3.52661 | 1.833245 | 6.784135 | 0.014241 | 1.68E-08 | 0.00016 |
| ROPN1 | 4.283675 | 1.892693 | 9.695113 | 0.022914 | 1.73E-08 | 0.000481 |
| EFHC1 | 70002.34 | 341.2956 | 14358015 | 0.006014 | 1.83E-08 | 4E-05 |
| LRPPRC | 8.21E+13 | 12001871 | 5.61E+20 | 0.006291 | 1.97E-08 | 6.61E-05 |
| TOGARAM2 | 4.128561 | 2.277649 | 7.483602 | 0.001254 | 2.72E-08 | 2.98E-06 |
| TEKT2 | 1.832459 | 1.372805 | 2.446017 | 0.005137 | 2.8E-08 | 3.95E-05 |
| BLOC1S6 | 9.25E+09 | 21501.63 | 3.98E+15 | 0.021978 | 2.8E-08 | 0.000526 |
| MECP2 | 143.8619 | 10.96176 | 1888.041 | 0.012196 | 3.33E-08 | 0.000155 |
| GSK3B | 39195959 | 1814.361 | 8.47E+11 | 0.024604 | 3.77E-08 | 0.000596 |
| CHMP2B | 1.32E+09 | 8214.353 | 2.13E+14 | 0.024604 | 3.77E-08 | 0.000596 |
| KIF21A | 86013.06 | 136.5506 | 54179515 | 0.023528 | 5.38E-08 | 0.00055 |
| NEK10 | 7.33E+08 | 74173.27 | 7.25E+12 | 0.004743 | 6.59E-08 | 1.37E-05 |
| SLAIN1 | 2.709683 | 1.705701 | 4.304612 | 0.002293 | 8.15E-08 | 2.43E-05 |
| DYNC2LI1 | 1701.729 | 39.54369 | 73232.5 | 0.009968 | 9.28E-08 | 0.000106 |
| RHOT1 | 104645.6 | 127.6016 | 85819392 | 0.026347 | 9.93E-08 | 0.000734 |
| SEPTIN1 | 2.56E+15 | 1.25E+08 | 5.23E+22 | 0.002923 | 1.08E-07 | 3.62E-05 |
| SPRY2 | 1.66E+21 | 6.54E+11 | 4.22E+30 | 0.00201 | 1.14E-07 | 9.77E-06 |
| MAP9 | 72.05068 | 7.693376 | 674.7753 | 0.012604 | 1.21E-07 | 0.000179 |
| HDAC3 | 2.75626 | 1.744276 | 4.355373 | 0.003943 | 1.27E-07 | 1.4E-05 |
| OPA1 | 139.9139 | 13.55043 | 1444.669 | 0.003423 | 1.33E-07 | 3.35E-05 |
| CELSR2 | 6.17E+10 | 615476.2 | 6.18E+15 | 0.00198 | 1.48E-07 | 2.35E-05 |
| FBXO5 | 1261.617 | 39.01164 | 40800.09 | 0.007203 | 1.5E-07 | 5.68E-05 |
| MAP7 | 1.855989 | 1.3701 | 2.514194 | 0.005451 | 1.56E-07 | 6.52E-05 |
| DYNLT1 | 43663564 | 9503.947 | 2.01E+11 | 0.004644 | 1.71E-07 | 4.33E-05 |
| TACR1 | 1.35E+13 | 7908919 | 2.31E+19 | 0.004227 | 1.73E-07 | 3.64E-05 |
| SPICE1 | 76209.59 | 1108.247 | 5240622 | 3.82E-05 | 1.8E-07 | 1.91E-07 |
| SPAG16 | 10538.82 | 248.6565 | 446667.3 | 0.000302 | 2.1E-07 | 1.26E-06 |
| DLG1 | 9971.275 | 91.30452 | 1088953 | 0.007794 | 2.16E-07 | 0.00012 |
| CEP97 | 50703101 | 7416.185 | 3.47E+11 | 0.008488 | 2.4E-07 | 8.22E-05 |
| WDPCP | 14333573 | 855.8344 | 2.4E+11 | 0.028164 | 2.42E-07 | 0.000898 |
| PARP3 | 89460.06 | 247.3841 | 32350913 | 0.007924 | 2.44E-07 | 0.000148 |
| CAPN6 | 3.69E+28 | 8.47E+12 | 1.61E+44 | 0.019971 | 2.56E-07 | 0.000343 |
| TTC21A | 2.47E+20 | 4.7E+10 | 1.3E+30 | 0.002708 | 2.7E-07 | 3.93E-05 |
| CFAP73 | 199.543 | 9.626972 | 4136.026 | 0.023221 | 2.77E-07 | 0.000617 |
| PURA | 310.0287 | 16.29041 | 5900.268 | 0.008169 | 2.89E-07 | 0.000135 |
| EML1 | 1.72E+19 | 7.18E+09 | 4.11E+28 | 0.003594 | 2.9E-07 | 5.83E-05 |
| ATF5 | 1.74E+14 | 8303751 | 3.65E+21 | 0.007817 | 3.04E-07 | 0.000138 |
| XPO1 | 3.476067 | 2.226767 | 5.426271 | 0.000118 | 3.15E-07 | 4.18E-08 |
| CKAP5 | 111.3659 | 9.563013 | 1296.909 | 0.012423 | 3.2E-07 | 0.000168 |
| CDK2 | 5.165363 | 2.650745 | 10.06546 | 0.000262 | 3.92E-07 | 1.41E-06 |
| ROPN1L | 24267259 | 8628.372 | 6.83E+10 | 0.002647 | 4.35E-07 | 2.71E-05 |
| KIFAP3 | 757604.7 | 1157.388 | 4.96E+08 | 0.004475 | 4.87E-07 | 4.27E-05 |
| RNF4 | 45.80405 | 5.98992 | 350.2569 | 0.016657 | 4.89E-07 | 0.000229 |
| KLHL42 | 1.46E+38 | 1.43E+20 | 1.5E+56 | 0.005206 | 4.98E-07 | 3.28E-05 |
| GADD45A | 21.85422 | 3.97743 | 120.0792 | 0.018651 | 5E-07 | 0.000388 |
| NME7 | 2.724963 | 1.731498 | 4.288441 | 0.001326 | 5.01E-07 | 1.47E-05 |
| PARD6A | 19.33453 | 3.883756 | 96.25321 | 0.020241 | 5.22E-07 | 0.000298 |
| PEX14 | 71967.2 | 87.84241 | 58961020 | 0.030002 | 5.38E-07 | 0.001085 |
